# Male African Elephant (*Loxodonta africana*) Behavioral Responses to Estrous Call Playbacks May Inform Conservation Management Tools

**DOI:** 10.3390/ani12091162

**Published:** 2022-05-01

**Authors:** Caitlin E. O’Connell-Rodwell, Monica N. Sandri, Jodie L. Berezin, Jaquelyn M. Munevar, Colleen Kinzley, Jason D. Wood, Maggie Wiśniewska, J. Werner Kilian

**Affiliations:** 1 Center for Conservation Biology, Stanford University, Stanford, CA 94305, USA; 2 Utopia Scientific, P.O. Box 221100, San Diego, CA 92192, USA; mnsandri@ucdavis.edu (M.N.S.); jberezin@smith.edu (J.L.B.); 3 Harvard University Center for the Environment, Cambridge, MA 02138, USA; 4 Geography Graduate Group, University of California, Davis, Davis, CA 95616, USA; 5 Department of Biological Sciences, Smith College, Northampton, MA 01063, USA; 6 Department of Surgical and Radiological Sciences, School of Veterinary Medicine, University of California, Davis, Davis, CA 95616, USA; drjaquelynmunevar@gmail.com; 7 Conservation Society of California, Oakland Zoo, Oakland, CA 94605, USA; colleen@oaklandzoo.org; 8 SMRU Consulting, Friday Harbor, WA 98250, USA; jw@smruconsulting.com; 9 The Federated Department of Biological Sciences, New Jersey Institute of Technology, Newark, NJ 08901, USA; mw298@njit.edu; 10 Etosha Ecological Institute, Ministry of Environment, Forestry, and Tourism, Okaukuejo via Outjo P.O. Box 6, Namibia; werner.etosha@gmail.com

**Keywords:** African elephant, estrous call playback, acoustics, mitigation tool, musth, human–elephant conflict, Namibia

## Abstract

**Simple Summary:**

During annual periods of heightened sexual activity (musth), male African elephants expend a significant amount of energy communicating their reproductive status, as well as searching and competing for potential mates. To locate females, musth males may risk venturing outside protected areas and into landscapes shared with humans where conflict between wildlife and people can occur. Adverse interactions between elephants and people can be detrimental to human livelihoods, resulting in negative attitudes towards elephants and, in some cases, retaliatory killings. Interactions with aggressive musth males can also be life-threatening to community members who attempt to confront them. Mitigation strategies that effectively target the reproductive motivations of musth males may offer solutions as the human–elephant interface continues to expand. In this study, we build on earlier research showing that playbacks of female elephant reproductive calls, (i.e., estrous rumbles) can change the movement trajectory and behavior of male elephants in Etosha National Park, Namibia. Individuals belonging to three male groups were opportunistically subjected to playback experiments and evaluated based on their reaction intensity. Our results demonstrate that mature musth adults are more likely to change directions and approach the source of a female’s estrous call than mature, sexually-inactive adult elephants. We also show that post-dispersal young males that were not in musth also respond strongly to the stimulus. These findings support further exploration of mitigation solutions that incorporate elephant behavior, reproductive status, and context-specific vocalizations.

**Abstract:**

Driven by reproductive motives, male African elephants (*Loxodonta africana)* in musth often expand their home ranges to locate estrous females. This extended range, coupled with heightened aggression often observed in musth males, can be particularly problematic in regions where human-modified landscapes and elephant territories increasingly overlap. Several mitigation tools have been tested to resolve a wide range of human–elephant conflicts with varying degrees of success due to geographical disparities and habituation. We present findings on the potential application of estrous call playbacks in manipulating the behavior and movement of male elephants non-invasively, particularly mature musth adults and younger post-dispersal males, in Etosha National Park. Estrous vocalizations were presented across 26 experimental trials to mature musth adults (*n* = 5), mature non-musth adults (*n* = 6), and non-musth males belonging to younger, post-dispersal age classes (*n* = 8), with behavioral responses scored on a gradient scale from 0–1. Both mature musth adults and younger non-musth elephants were significantly more likely to respond with the highest intensity by approaching the acoustic source compared to mature non-musth adults that avoided the call. However, younger males tested in the presence of an older, higher-ranking male tended to react with a lower intensity than those tested alone. This result likely demonstrates the influence of social hierarchy and associations on male elephant behavior. We also observed a significant increase in physiological response, measured by defecation rate, across all male groups in response to the estrous call playbacks. Our findings suggest that using estrous calls as acoustic deterrents may effectively and non-invasively aid in reducing tension at the human–elephant interface, depending on the age, social context, and reproductive status of the male elephant.

## 1. Introduction

Male reproductive success in polygynous and sexually dimorphic species is often driven by intense intrasexual competition [1,2] and associated with risk-taking behavior [3,4,5,6]. In order to achieve maximum reproductive potential and compete for access to receptive females in estrus, males require physiological and behavioral strategies that enhance sexually dimorphic traits, increase body size, and improve dominance rank [7,8,9]. In African elephants (*Loxodonta africana*), adult males experience an annual, asynchronous period of heightened aggression and sexual activity known as musth [2,10,11,12,13]. This hormonal state is marked by pronounced physiological symptoms and behaviors that communicate status and increase reproductive success [2,11,14].

As male elephants come into musth, they expand their ranges in search of estrus females within highly mobile family groups [7,15,16]. Although female elephants are considered more risk-averse than males and prefer to avoid human-modified landscapes (HMLs) when possible [17,18], females do crop-raid in some systems [19,20], and many family groups utilize travel corridors in unprotected landscapes [21,22,23,24]. Moreover, family groups occupy areas beyond the confines of wildlife preserves all across the African continent [16,17,22,25]. These factors drive musth males to travel farther, faster, and with more directional purposes [11,26,27,28,29,30]. As a result, elephants that are sexually active or in musth may enter unprotected areas adjacent to wildlife parks where human–elephant conflict (HEC) continues to intensify [20,31,32].

HEC represents a wide range of antagonistic interactions between humans and elephants that can be both indirect (e.g., resource competition and fear of conflict) [33] and direct (e.g., crop-raiding and the destruction of personal property, such as water installations and grain houses) [3,6,33,34,35,36,37]. Fence-breaking is an equally common and problematic form of HEC that has considerable corresponding costs and counteracts exclusionary mitigation methods [32,38]. Male elephants can often easily circumvent or break through physical barriers in order to gain access to vital resources during non-musth phases and periodically in their attempt to locate reproductively-active estrous females beyond protected areas during their musth period [6,34,37,38,39].

As a result, elephants discovered outside fenced reserves, such as Etosha National Park, Namibia, can fall victim to retaliatory killings by game wardens and community members [40,41,42,43,44] and are occasionally chased back into the park using helicopters. Such tactics can be exceedingly stressful, costly, and have the potential to harm both elephants and people involved, as well as other wildlife in close proximity. In addition, these measures only offer short-term relief and could ultimately result in further escalation and injury to elephants and other wildlife [32,33,36,45]. If left unresolved, negative interactions between elephants and people can become chronic [20,32,46]. Therefore, it is critical to establish effective non-invasive solutions to HEC, particularly in landscapes adjacent to protected wildlife areas where the human–elephant interface continues to expand [20,36,47,48,49].

Since elephants are highly intelligent, social mammals that develop vast repositories of social and referential knowledge over their long lifetimes [50,51,52], HEC mitigation strategies that integrate bioacoustic tools warrant further investigation. Elephants can produce and differentiate between a wide range of context-specific vocal cues, and demonstrate unique behavioral responses to a variety of acoustic stimuli, such as the sound of disturbed African honeybees (*Apis mellifera scutellata*) [50,53,54], human voices from different ethnic groups [50,55], and antipredator calls [20,50,51,56]. Previous studies have demonstrated that elephant behavior can be manipulated using acoustic stimuli associated with danger, thereby eliciting a fear response and resulting in avoidance of the sound source [20,57]. However, fear-based acoustic cues may be less effective in the long-term for HEC mitigation, particularly for males, since elephants can habituate to deterrents that pose no real threat and, at least in some cases, show no response at all [20].

Targeting male elephant reproductive motivations using female estrous vocalizations may serve as an effective alternative to fear-based acoustic tools currently implemented for HEC mitigation, depending on regional patterns of musth male movement and occurrence across HMLs. Adult female elephants only enter into estrus every four-five years due to lengthy gestational and postpartum lactation periods that prolong inter-calving intervals [12,58,59]. Moreover, peak estrus in female African elephants only lasts two–three days [27,60], making it imperative that males recognize a female’s reproductive status and locate her across vast landscapes. To signal their reproductive status and attract males, estrous females engage in distinctive behaviors [58], emit characteristic estrous roars when chased [61] and, during peak estrus, produce a series of low-frequency estrous rumbles immediately after mating [27,30,52,62]. These low-frequency estrous calls can propagate on the order of several kilometers and are likely to be heard by many elephants [56,63].

In two field studies where prerecorded estrous rumbles were presented to male elephants of different reproductive statuses, results showed that musth elephants tend to approach the sound source, whereas older non-musth (sexually inactive) individuals tend to listen and flee [56,63]. This was the case even when calls were played from a distance of 1.2 kilometers (km) away [56]. These findings suggest that, depending on the age and reproductive motivations of the target subject and the meaning of the vocalization employed, it may be possible to non-invasively manipulate elephant behavior and reduce HEC using context-specific acoustic tools.

The aim of this study was to test the efficacy of a reproduction-specific acoustic stimulus at altering the movements of post-dispersal male elephants in Etosha National Park, Namibia, with implications for HEC mitigation. Since musth males are almost exclusively focused on locating and mating receptive females, we predicted that estrous call playbacks may effectively attract musth males toward the sound source and away from their intended path of movement. If their path could be influenced, such playbacks could serve as a tool to steer musth males away from potential conflict areas and back into the park. Likewise, we anticipated that non-musth males yet to reach their reproductive prime (≤34 years old) would demonstrate a heightened interest in the estrous call. In contrast, we hypothesized that mature non-musth adults would not respond or would promptly leave the area, possibly to avoid potential conflict with competitive musth males nearby. We also explored whether the sudden onset of an estrous call playback would trigger an increase in the rate of defecation as further evidence of a conditioned evolutionary reaction in the context of mating. Lastly, we present movement data from five resident male elephants in order to show that males do travel beyond park boundaries, mostly during their expanded wet season range, but also during musth. This highlights the potential need to mitigate the risk of negative interactions between humans and male elephants, including adults in musth, throughout our region of study.

## 2. Materials and Methods

### 2.1. Study Site

**Figure 1:**
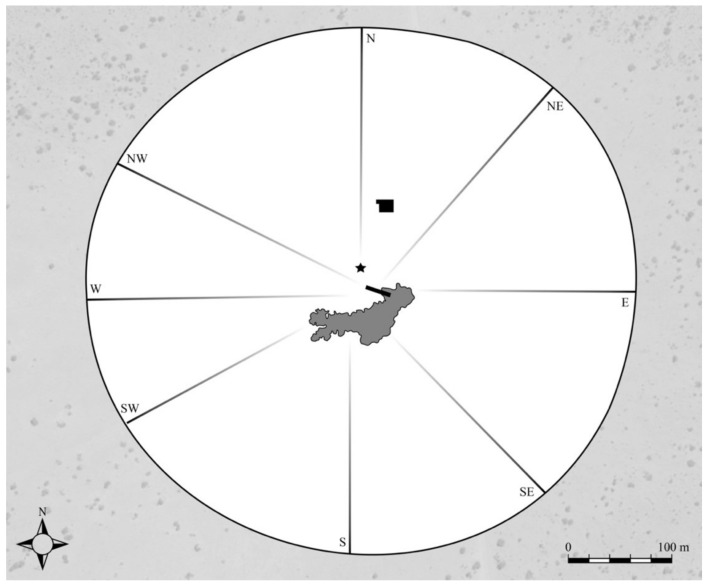
Map of the Mushara waterhole and surrounding 0.22 km^2^ clearing, located in the northeastern corner of Etosha National Park, Namibia. The center of the map is marked by a water trough (rectangle shape), which is filled by an artesian well that is controlled by a ball valve. The water spills out from the east end of the trough, creating a small pan (in gray). Approximately 80 m north of the trough is the 8 m observation tower where researchers were positioned during playback experiments. The star on the map marks the location of the hidden speaker used to broadcast the estrous vocalization remotely from the tower. The vocalizations are presented to male elephants as they reach the edge of the clearing (perimeter circle). Major elephant pathways to and from the waterhole are depicted and directions labeled.

Playback experiments were conducted during the 2008 dry season and once again in 2010 at the remote Mushara waterhole (Figure 1), located in the northeastern corner of Namibia’s Etosha National Park (ENP). ENP is roughly 22,970 km² [64] and supports an elephant population of approximately 2900 individuals within its fenced borders [65]. Elephants do, however, circumvent or break through park fence lines and travel between ENP and HMLs surrounding the park [66,67].

In addition, this semi-desert region is characterized by an annual wet season from November to April and dry season from May to October [68]. Years are further categorized as “wet” or “dry” based on annual rainfall collected during the preceding wet season at the nearby Namutoni weather station. Namutoni’s long-term mean annual rainfall is 436 mm, with mean rainfall measuring 400 mm in dry years and 650 mm during wet years ([64,67,68], Etosha National Park rain data archive, unpub. data). Both 2008 and 2010 were categorized as relatively wet years (600 mm and 585 mm, respectively), which tend to correspond with higher numbers of musth males and smaller group sizes of males [68].

The Mushara waterhole is fed by a permanent, artisanal spring and is the only stable source of drinking water within a 10 km radius [68,69]. It is a critical resource to local wildlife, particularly during the dry season months when ephemeral water dries up. Behavioral observations were collected from an 8 meter (m) tower situated 80 m from the waterhole and with a full view of the surrounding 0.22 km² clearing (Figure 1). Male elephants come to the waterhole to drink during the daytime and throughout the night, while family groups often arrive at or after dusk up until about 2 a.m. [70].

### 2.2. Elephant Identification, Age Classification, and Musth Status

In 2008, all post-pubertal male elephants (*n* = 20) that visited the waterhole alone or in groups of two were opportunistically subjected to playback experiments (mean trials per individual = 1.37 ± 0.76). As part of a long-term monitoring project of male elephants since 2004, known individuals were identified using recognizable morphological traits, (e.g., ear tear patterns, tail hair configuration, tusk size and shape, and overall body size) and assigned a relative age class based on shoulder height and hindfoot lengths [68,69,71]. Age classes of male elephants include: one quarter (1Q), or 10–14 years old; half (2Q), or individuals between 15–24 years old; three quarter (3Q), or males between 25–34 years in age; and fully mature (4Q) adults, approximately 35 years and older (Table 1).

In addition, musth status was evaluated based on the presence of musth-specific traits, such as active urine dribbling, urine staining on the back legs, temporal gland swelling, and secretions [11,12,61,72]. Behaviors known to be associated with musth—such as ear waving, trunk curling, trunk dragging, and the musth walk—were also recorded [12,61,72,73]. Elephants were considered in musth if a suite of these visible signs were displayed during behavioral observations. Although younger musth males, (i.e., ≤3Q age class) are observed in this population on occasion, only musth elephants in the 4Q age class were encountered during the 2008 field season. Furthermore, mature 4Q males are considered prime breeders, given the competitive advantage of their larger body size and longer-lasting musth periods (up to 10 weeks) in comparison to younger males (a few days to a couple of weeks) [2,12,27]. As such, all males within the 4Q age class were divided into musth and non-musth categories, while males of 3Q age or younger were combined to form a single group of non-musth individuals that have yet to reach their competitive, reproductive prime.

Due to patterns of sporadic visitation to the Mushara waterhole during wetter years when ephemeral pools provide additional places to drink [69], the population of potential test subjects was much reduced. Nonetheless, five musth 4Q males, six non-musth 4Qs, and eight younger individuals in the non-musth 1Q–3Q male group were identified and presented with estrous call playbacks in 2008 across 26 trials (Table 1). Five of these individuals were observed at the waterhole more than once and were thus subjected to multiple playback experiments to explore the potential for habituation with repeated exposure to the stimulus. The mean number of trials these individuals participated in ranged from two to four, with a mean inter-trial time period of 7.14 ± 4.3 days. One musth 4Q individual tested in 2008 was also presented with estrous call playbacks on a single occasion in 2010 to evaluate changes in response over varying environmental and hormonal conditions, as well as the potential of habituation.

### 2.3. Estrous Call Playback Protocols

**Figure 2:**
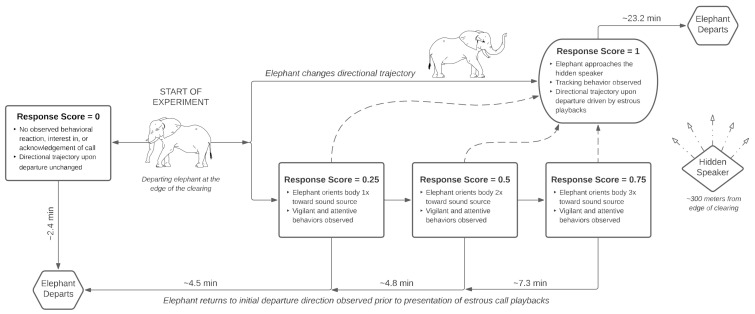
Flowchart of the estrous playback experimental design and suite of responses observed from *n* = 19 male African elephants. Upon departure from the Mushara waterhole clearing, males are presented with a prerecorded estrous rumble once per minute until (1) the elephant begins to track the call, (2) the social environment changes, or (3) the elephant leaves the clearing. Response criteria are based on the presence and frequency of focal behaviors described in Table 2. Elephant responses of ≤0.75 departed in the same direction observed prior to playback presentation, whereas the departure direction of individuals with a score of 1 was dependent on the elephant’s position to the hidden speaker. Departing and Periscope Elephant Graphics © 2022 Monica Sandri.

Upon arriving at the waterhole, male elephants were given the opportunity to drink and interact with conspecifics before being presented with estrous call playbacks. Trials began when lone or paired males were leaving the clearing and approximately 300 m away from the hidden speaker (Figure 2). A single estrous rumble exemplar was then broadcasted once per minute (min) until the focal subject(s) chose to depart or approach the stimulus source.

Although the timing and frequency of calls presented do not replicate that of a real estrous female’s vocal behavior (typically given as a series of rumbles [62]), the time male elephants visited and spent at the waterhole was very brief due to wet year conditions. As such, we opted to maximize our chances of observing and interpreting a behavioral response. To minimize potential habituation to the call, test events were terminated when subjects moved beyond the clearing’s edge or if a change in behavioral motivations was detected, (e.g., a new elephant enters the clearing or the test subject returns to the water trough and drinks for ≥5 min).

We broadcasted previously recorded estrous rumbles provided by Dr. Joyce Poole from two female elephants in Amboseli National Park, Kenya [62]. Although estrous calls from two different females were provided, preliminary results indicated a preference for one of the callers. As such, we used the estrous call from a single individual to maximize a potential response, due to the limited opportunity for presentation during a wet year. Further information regarding collection protocols and estrous call identification can be found in the aforementioned 1988 publication. Vocalizations were played from a low-frequency Panasonic speaker (Panasonic Corporation, Osaka, Japan) capable of broadcasting the signal with a fundamental frequency (F_0_) of ±17–20 Hz [70]. The speaker was hidden approximately 20 m distance from the waterhole and 300 m from the edge of the clearing (Figure 1). To resemble the natural amplitude of an elephant call (120 dB SPL at 1 m, as measured by a decibel meter), the bioacoustic stimulus was further amplified with a 1000 W amplifier (see O’Connell-Rodwell et al. 2007 for further information). In addition, an Apple iPod music player was used to remotely control the delivery of the exemplar.

### 2.4. Response Scores and Behavioral Data Collection

Elephant behavioral responses were first scored in situ by an expert in elephant behavior (C.E.O.) on a gradient scale from 0 to 1, based on a percent reaction intensity criteria established prior to experimentation (Table 2 and Figure 2). A score of 0 (0%) was assigned if target males exhibited no apparent reaction to the call and continued on their directional trajectory out of the clearing. By contrast, a score of 1 (100%) represented the greatest response intensity and was characterized by males tracking [61,73] and approaching the speaker. If males reacted to the call by immediately freezing, listening, and/or orienting their entire body once towards the direction of the sound (vigilant and attentive behaviors, see Table 2) [61,73,74], they were considered to have a response intensity of 25% (score = 0.25). This score represents the minimum reaction to the estrous call, rather than to another potential sound or environmental stimulus. Scores of 0.5 (50%) and 0.75 (75%) were recorded in elephants that demonstrated a stronger interest than exhibited in the 0.25 response, measured by the number of additional orientations towards the acoustic source without an observed approach (Figure 2). Behaviors in the context of social cohesion, avoidance, and attraction (except for musth displays; Table 2), as well as the total duration elephants remained in the clearing after playback presentation, were considered forms of decision-making in elephants. All other observed focal behaviors fell under the context of conditioned evolutionary reactions elicited by the call.

Additionally, a uninformed secondary observer (C.K.) independently collected behavioral data of male elephants during estrous call experiments in a Noldus, The Observer^®^ datalogger (version 5, Wageningen, The Netherlands) [75] using a continuous, all-occurrence sampling method. Elephant expert C.K. was not made aware of the response score criteria implemented by C.E.O. so as to prevent observation bias. A trained third-party (M.N.S.) ranked data recorded by C.K. that was entered into a Noldus behavior datalogger and independently scored the responses of male elephants subjected to estrous playback experiments, given the reaction intensity criteria and ethogram of focal behaviors (Table 2 and Figure 2). In this way, the interpretation of behavioral responses and associated scores were evaluated across multiple observers.

In all, 19 males of known age were subjected to estrous playback experiments across 21 testing events for a total of 26 trials (1.37 ± 0.76 mean number of trials per individual). Three testing events contained two elephant subjects, and so were simultaneously presented with playbacks. Prior to statistical analyses and subsequent comparisons, a mean response score was calculated for each male that participated in more than one testing event (*n* = 5) in order to account for uneven repeated measures and equally weigh scores between individuals. Datalogger records were also used to calculate the duration of time elephants remained in the clearing after exemplar broadcasting and summarized by median (±IQR) time in minutes (min) across trials and response scores.

Lastly, we assessed whether the rate of two relevant behavior categories (physiological and musth) changed between two time windows (total time before and after the onset of the first estrous call). This allowed for behavioral comparisons across elephants while controlling for differences in total observation time between individuals. Using the behavioral data collected for all subjects that had arrival and departure timestamp records (*n* = 18), “before” period observation times were measured in minutes from an elephant’s arrival timestamp to the start of playback trial experiments, and then “after” from the timestamp of the first exemplar broadcast to the timestamp of experiment termination.

Defecation rates were determined for males by summing the observed count per time period and then dividing by the “before” and “after” observation times, respectively. Similar procedures were used to calculate the rate of combined musth behaviors observed in mature musth adults, including the following behaviors: trunk drag, ear wave, musth walk, trunk curl, and tusking the ground [2,10,11,12,61,73]. Other musth behaviors, such as urine dribbling and temporal gland secretions, were excluded given their often continuous and categorical nature. Rates acquired for individuals subjected to multiple playback trials in 2008 were then averaged so that all participants had an equivalent number of rates for each time period prior to conducting analyses of rate comparisons.

### 2.5. Male Elephant Movement

In October of 2009, five resident male elephants of post-dispersal age (elephant ID #265 in the 2Q age class, and ID #264, 266, 267, and 268 in the 4Q age class) were fitted with Global Positioning Satellite (GPS) and Global Systems for Mobile Communications (GSM) collars by the Namibian Ministry of Environment and Tourism in order to evaluate elephant movement patterns across ENP [66]. Data collection occurred over a two-year period, with positional information recorded every 15 minutes. To express tracking data in conventional metric units, we used the Universal Transverse Mercator coordinate system (UTM) projection [76].

We analyzed the following parameters with this dataset: (1) elephant presence in regions beyond park boundaries, including adjacent livestock HMLs and agricultural HMLs north of the Omuthiya road, (2) patterns of seasonal movement outside ENP, and (3) movement through nearby HMLs that coincided with musth periods of collared males. Males were evaluated for musth during behavioral observations at the Mushara waterhole, based on the presence of musth-specific traits (see Section 2.2. Elephant Identification, Age Classification, and Musth Status). Given that our field season does not always cover an elephant’s entire musth period, the duration of musth in collared 4Q males was estimated as three months, based on average durations documented in other populations [7,77,78].

### 2.6. Statistical Analyses

All statistical analyses were performed in R statistical software (version 4.0.3) [79] using an alpha level of α = 0.05.

To assess the agreement between elephant behavioral response datasets generated by two independent rankers (Table S1), an intraclass correlation coefficient (ICC) was calculated with the ‘icc’ function in the R package *irr* (version 0.84.1) [80] using a two-way random-effects model with an absolute agreement type [81,82]. Given a high inter-rater reliability score (ICC = 0.91, 95% CI = 0.75–0.97), response scores were averaged across raters. A one-way ANOVA was then performed to determine if there was a significant difference in the mean behavioral response score between male groups (Table S2). Post hoc pairwise comparisons were conducted using a Tukey’s HSD test (95% CI) to determine which groups were different from each other.

We also assessed whether male elephants in our study exhibited physiological reactions to estrous call playbacks in the time period after the broadcast. In this analysis, we compared defecation rates observed in the time periods “before” and “after” estrous playbacks. A non-parametric exact Wilcoxon signed-rank test [83,84,85] was performed using the ‘wilcox.test’ function in the R package *stats* [79] with the alternative parameter, “less,” to evaluate whether the rate of defecation increased significantly upon exposure to the estrous call playbacks (Table S3). Similarly, an exact Wilcoxon signed-rank test was also used to evaluate the difference between musth behavior rates calculated for males in musth (*n* = 5) during the time periods “before” and “after” playbacks (Table S4). A second Wilcoxon test was then run for comparison and only included musth 4Q males that had a reaction score of 1 (Table S5).

## 3. Results

### 3.1. Response Scores and the Duration of Behavioral Responses

**Figure 3:**
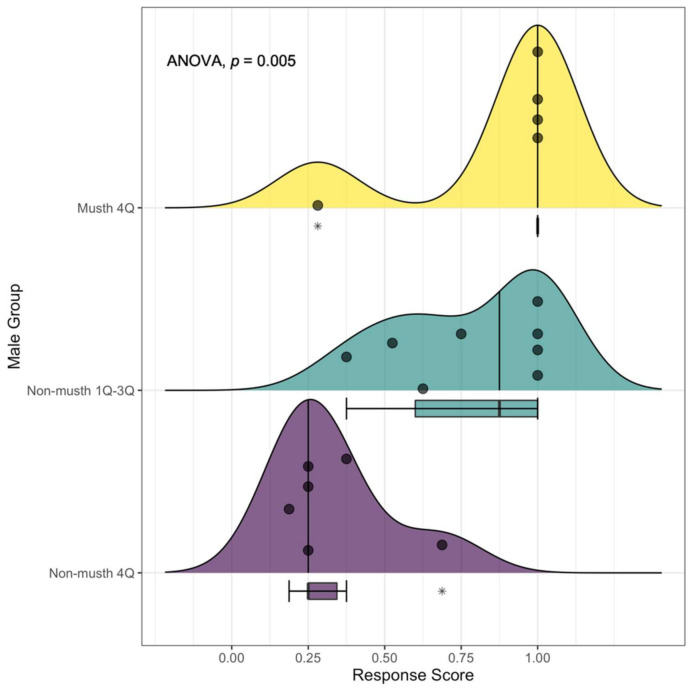
The distribution of behavioral response scores observed in male African elephants during estrous playback experiments at the Mushara waterhole in 2008. For each male elephant group, ridgeline peaks showcase the density of elephants, represented by individual points along the response score gradient. Median values (non-musth 1Q–3Q = 0.875; non-musth 4Q = 0.25; and musth 4Q = 1.00) are shown using a vertical line per group. Corresponding boxplots demonstrate interquartile ranges (non-musth 1Q–3Q = 0.40; non-musth 4Q = 0.094; and musth 4Q = 0) and outliers (*). The boxplot for the musth 4Q group is compressed to a single line below the median due to the heavily weighted response score of 1, with a single extreme outlier present at 0.28. The effect of male group on response score was significant (*p* = 0.005).

As expected, male elephants in the musth 4Q group produced the highest mean response score (0.86 ± 0.32) when presented with estrous call playbacks across nine testing events (Figure 3). Four out of five of these test subjects (80%) exhibited a mean response score of 1 (Figure 3), characterized by searching and approaching the sound of the female vocalization (Figure 2 and Video S1). However, a single musth male demonstrated avoidant behaviors during each of his four playback trials, resulting in an average individual score of 0.28 ± 0.04 (Figure 3). In contrast, this same elephant was subjected to playback experiments in 2010 during his annual musth period and exhibited behavioral reactions similar to those observed in all other musth males during 2008, (i.e., mean response score = 1).

The average response score of the non-musth 1Q–3Q group was also high (mean score = 0.78 ± 0.25), with four out of eight individuals (50%) yielding a score of 1 (Figure 3). Two of three males in the younger demographic group (both in the 3Q age class) were subjected to estrous playbacks in the presence of a dominant 4Q male and responded by following the dominant individual away from the sound source, whereas the third male (1Q in age) chose to approach the sound source instead of moving in the direction of the dominant male. By contrast, the group of six non-musth 4Qs exhibited the lowest response of any group (mean score = 0.33 ± 0.18), with no individual yielding a mean response score greater than 0.69 (Figure 3). Therefore, all non-musth 4Q elephants subjected to reproduction-specific acoustic cues reacted by avoiding the call and leaving the waterhole (Figure 2).

One-way ANOVA results indicated that mean response scores were significantly different across male groups (*F*(2,16) = 7.43, *p* < 0.005; Table 3 and Figure 3). Additionally, post hoc pairwise comparison tests revealed a significant difference between the response scores of non-musth 4Qs and musth 4Q elephants (*p* = 0.009), as well as between the non-musth 1Q–3Q group and non-musth 4Qs (*p* = 0.012). No significant difference in response was observed between musth 4Qs and the non-musth 1Q–3Q group (*p* = 0.874).

Median durations (±IQR) calculated across trials showed a definite increase in the amount of time elephants spent at the clearing as response scores increased (Figure 2). Only one male had a response score of 0 on one occasion and remained in the clearing for the shortest amount of time (2.35 min). Males who had a response score of 0.25 and 0.5 were found with similar durations (4.5 ± 1.52 and 4.75 ± 1.21 min, respectively), while elephants with a score of 0.75 spent approximately 7.34 ± 1.87 min in the clearing prior to departure. Elephants that were observed with the highest behavioral response of one spent substantially more time at the Mushara waterhole clearing after the first exemplar broadcast (23.2 ± 11.72 min). Further exploration revealed that musth 4Qs with a response score of one consistently spent more time searching for the sound source compared to non-musth 1Q–3Q individuals with the same score (23.2 ± 6.28 and 4.86 ± 12.71 min, respectively).

### 3.2. Comparing Rates of Defecation and Musth Behaviors across Time Periods

**Figure 4:**
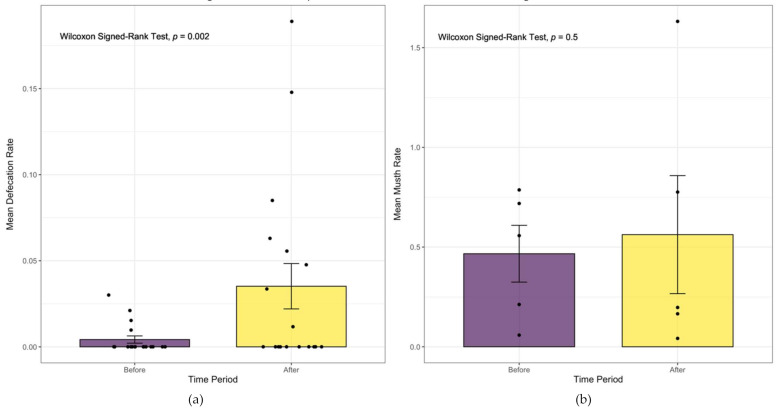
(**a**) The mean rate (±SEM) of defecation per minute observed in male elephants (*n* = 18) “before” and “after” the onset of estrous call playbacks. Individual elephants are represented by a single point for each time period and are jittered along the categorical x-axis to prevent point overlap. No difference was detected between elephants with divergent reaction scores. There was a significant difference between before and after values (*p* = 0.002, effect size r = 0.65); (**b**) the mean rate (±SEM) of musth behaviors per minute observed in all mature musth adult elephants (*n* = 5) across both time periods. There was no significant difference between musth behavior rates observed across both time periods (*p* = 0.5, effect size r = 0.06).

Although the mean rate of defecation observed in all male elephants was low, we found a significant difference between the “before” (0.004 ± 0.01) and “after” (0.035 ± 0.06) testing periods (Wilcoxon signed-rank test *p* = 0.002, effect size r = 0.65, magnitude = large; Figure 4a). In addition, the mean rate of musth behaviors slightly increased in the time period after testing, but not significantly so (“before” 0.47 ± 0.32 and “after” 0.56 ± 0.66; *p* = 0.5, effect size r = 0.06; see Figure 4b). When we tested only musth 4Q males with a mean response score of 1 (*n* = 4), similar trends were observed across both time periods (0.44 ± 0.36 and 0.66 ± 0.72, respectively; see Suppl R script) and were not significant (Wilcoxon signed-rank test, *p* = 0.31, effect size r = 0.37; see Figure S1).

### 3.3. Male Elephant Movement Patterns across Seasons and Reproductive States

**Figure 5:**
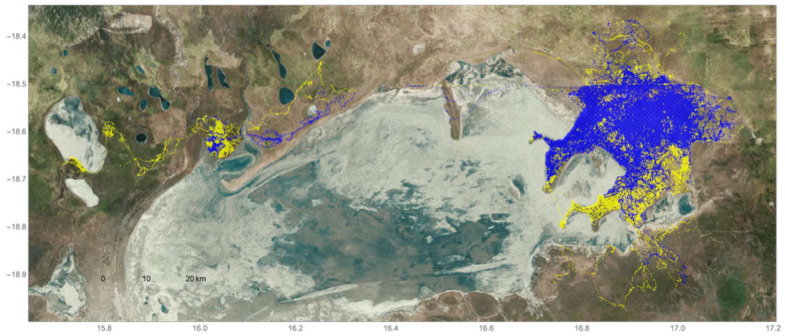
Movement data of male African elephants (ID #264, 265 266, 267, and 268) across the northeastern region of Etosha National Park (ENP), Namibia and collected between October 2009 and November 2011 using GPS collars. Elephant movement during the wet season (May–October) is showcased in blue and movements during the dry season (November–April) in yellow.

**Figure 6:**
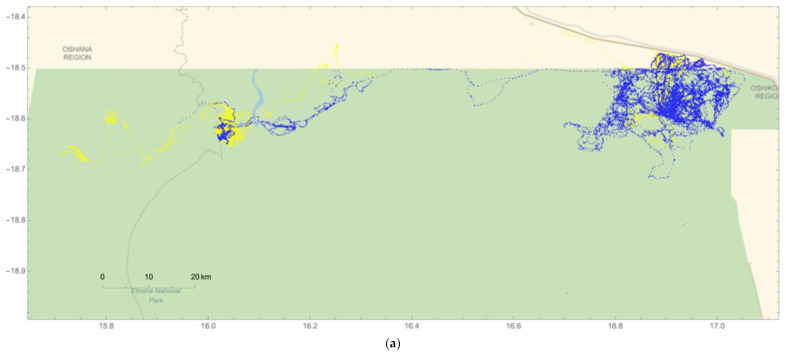

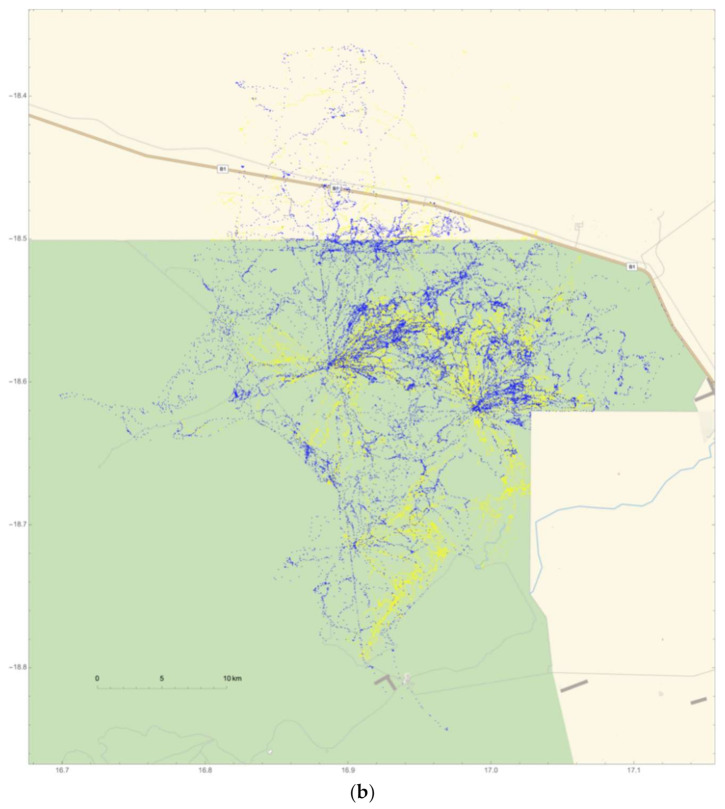
Two collared adult males (#264 and #267) traveled beyond park boundaries and into human-modified landscapes (HMLs) north of ENP during both wet and dry seasons. The edge of the green line in (**a**,**b**) represents the park’s boundary that abuts HMLs, shown in tan. Everything north of the Omuthiya road (brown line and labeled B1 on Figure 6b) is agricultural land where crop-raiding has been known to occur; (**a**) elephant #264 traveled across a large span of the northern HML region on a total of 166 days, while (**b**) #267 was observed in the northeastern HML region on a total of 76 days.

Of the five resident male elephants collared for this study, individuals #264 and #267 frequently moved into HMLs adjacent to park boundaries during both the wet and dry seasons (Figure 5 and Figure 6). In total, #264 was detected outside of the park on 166 days (Figure 6a), while #267 was observed beyond ENP on 76 days (Figure 6b). In addition, #264 traveled over the greatest span of the northern region of the park and abutting HMLs, but was not detected in agricultural areas above the Omuthiya road during the crop season in the wet season (Figure 6a). By contrast, #267 moved beyond livestock HMLs closest to the park and traversed the road into agricultural HMLs during both seasons (Figure 6b). Elephants #265, 266, and 268 all remained inside the park during both seasons for the entire two-year period (for individual movement patterns, please see Figure S2).

**Figure 7:**
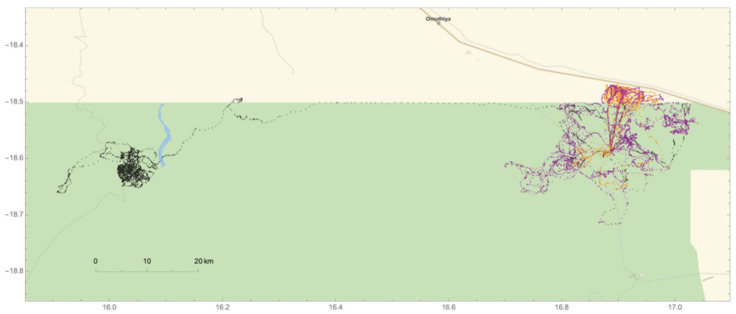
Movement patterns of adult elephant #264 across the northeastern region of Etosha National Park over a nine-month span in 2010. Black points represent movements during the three-month period before the onset of musth (May–July). Orange points represent #264′s movements during musth (31 July–31 October), and purple points depict movements during the post-musth period (November–January). During the post-musth period, #264 spends much of his time utilizing the entire northeast region of the park.

In addition, we were able to confirm that one of the collared adult males (#264) came into musth at the end of July 2010, based on observations of musth-specific traits at the Mushara waterhole. During the three-month period before entering musth (May–July), #264 spent the majority of his time in the northwestern region of ENP before traveling east along the northern boundary of the park (Figure 7). He was first observed at the Mushara waterhole on 20 July and demonstrated no outward signs of musth. On 30 July, #264 returned to the waterhole exhibiting a suite of musth behaviors. During #264′s projected three-month musth period that followed (31 July–31 October), he frequently traveled between the Mushara waterhole and non-agricultural HMLs (Figure 7). Post-musth movement from November–January revealed an expansion of movement across the northeastern corner of ENP, with some continued movement outside the park.

## 4. Discussion

This study demonstrates how context-specific acoustic stimuli can successfully elicit targeted behavioral responses in male African elephants, depending on age, reproductive motivations, and social context. Similar to earlier studies conducted in East Africa [56,63], we found that playbacks of estrous calls appealed to the reproductive motivations of mature musth elephants by attracting them away from their intended movement trajectory and toward the direction of the call. As expected, all but one musth male exhibited consistent tracking behavior over an extended duration of time after the onset of the first exemplar broadcast. This suggests that playbacks of estrous calls may serve as an effective tool when musth elephants venture into nearby HMLs and must be lured back into protected wildlife areas.

By contrast, mature non-musth adults consistently avoided the direction of the speaker while displaying vigilant and attentive behaviors. Males in this group also promptly departed from the testing arena, suggesting playbacks of estrous calls likely would not deter non-musth adults from their intended direction of movement. Interestingly, younger non-musth elephants in the 1Q—3Q group demonstrated heightened interest in the call but often refrained from approaching when in the presence of an older, more dominant male. This suggests that social dynamics and dominance hierarchies may be an important factor in decision-making when individuals are in all-male elephant groups, particularly for younger and lower-ranking individuals. Overall, estrous call playbacks show great promise as an HEC mitigation tool when targeting male elephants in musth as well as younger, lone males yet to reach their reproductive prime. This may be of particular value in regions where male elephant home ranges, including movement during musth, overlap with HMLs. Applications of playbacks for HEC mitigation are further elaborated upon below.

### 4.1. Factors of Decision-Making in Mature Adult Male Elephants

African elephants in the hormonal state of musth are primarily driven by reproductive motives, and thus allocate the majority of their time towards locating and competing for sexually-receptive females [11,12]. During this pursuit, mature individuals at peak reproductive capacity (≥35 years) experience dramatic weight loss during musth, and can also lose approximately 345 liters of fluid per day due to the expression of reproductive chemosignals, (i.e., urine dribbling and temporal gland secretions) [63,86]. This focused searching behavior is undoubtedly a major reason why musth adults in our study were more likely to change their intended movement trajectory toward the direction of the acoustic stimulus source, whereas non-musth 4Q males tended to freeze and display attentive behaviors prior to moving away from the hidden speaker. In addition, musth 4Q males with a response score of 1 were also observed tracking the acoustic stimulus over a prolonged period of time compared to all other male groups. These results are consistent with previous findings [56,63], and further signify the influence of motivational states on male elephant behavior. Moreover, our findings demonstrate how context-specific acoustic stimuli may elicit conditioned evolutionary responses, such as those observed when elephants are presented with the sound of swarming bees [50,51,53,54].

Non-musth adults, on the other hand, are predominantly motivated by energetic demands and spend the majority of their time foraging and resting in order to regain body condition post-musth [27,63,87]. As a result, non-musth adult elephants often choose to avoid potential risks and escalated contests with competitive musth males, particularly in the presence of estrus females [63]. Our findings support these outcomes and further demonstrate the complexity of male elephant decision-making, depending on motivational state and life history patterns [6,34]. As such, female estrous calls may be less suitable when the primary targets of regional conflict mitigation are non-musth adult males.

### 4.2. Young Adult Responses and the Potential Influence of Social and Environmental Conditions

Novel to this study, we show that estrous calls also attract younger males, which also has HEC implications. Consistent with our predictions, males in the non-musth 1Q–3Q group demonstrated an overall heightened level of interest in reproduction-specific acoustic stimuli, particularly when visiting the waterhole alone. Although juvenile and subadult males have not reached their reproductive prime, they frequently demonstrate an interest in estrus females, adopting ‘sneaking’ tactics in the presence of musth males, as well as chasing and attempting to mate with estrus females when older musth males are absent [27,58]. Given that male elephants are capable of producing sperm in quantity by age 17, a heightened interest in sexually active females may afford younger males the opportunity to accrue reproductive fitness benefits [88]. Elsewhere, competitively inferior young adults have been confirmed to sire a large proportion of a population’s offspring (29% Amboseli National Park [88] and 38% in Samburu/Buffalo Springs National Reserves [89]).

Other potential factors, such as environmental conditions at Mushara waterhole in 2008, likely contributed to the social environment and observed intensity of reactions of young adult males during estrous playback experiments. Looser dominance hierarchies have been observed in this population during years of high rainfall and can result in the increased display of aggression by subordinate males that are typically younger in age [69]. In addition, consecutive high rainfall years are well correlated with improved body condition, the occurrence of estrus, and the rate of conception in female elephants in East Africa [90]. Given the greater than average rainfall observed in Mushara during 2008 [68,69], it seems plausible that more reproductively receptive females may have also been available in 2008, perhaps prompting bolder behaviors in young adult males that had less oversight from older individuals given the increased water availability.

Interestingly, non-musth 1Q–3Qs tested in the presence of an older, more dominant individual either in or out of musth tended to respond to playbacks with a lower intensity than those tested alone. Younger male elephants were also observed following older conspecifics out of the Mushara clearing on all but one occasion. These findings indicate that the social environment might play a role in the assessment and decision-making of young adult males [69], particularly among 3Q males on the verge of entering their reproductive prime. Younger individuals, such as the rambunctious 1Q male that approached the sound source in the presence of an older musth male, are not yet viewed as a threat by mature males and are, therefore, less likely to be intimidated by their presence [27].

By contrast, individuals in the 2Q–3Q age range likely possess a greater understanding of the fitness consequences associated with investigating an estrous call in the presence of a larger, most likely more dominant male. As a result, these individuals might apply their knowledge of the social ‘status quo’ and adjust their behaviors accordingly [91,92]. Alternatively, low-scoring young adult males in our study may not have been in their sexually active period, thereby showing a greater interest in associating with other males rather than females [12,13,78]. As such, female reproductive vocalizations might serve as a valuable tool in attracting lone, post-dispersal males in the 1Q–3Q age group away from HMLs.

### 4.3. Divergent Responses over Time in One Musth Male

One mature musth 4Q elephant that was tested four times over the course of the 2008 field season exhibited behaviors of disinterest and promptly left the waterhole after estrous call presentation. This was unexpected, given the consistent outward behavioral and physiological signs of musth he exhibited throughout the season and the strength of response displayed by all other musth adults. In addition, male elephants typically experience a ‘ramping up’ period, characterized by a rapid increase in testosterone levels between the pre-musth and musth periods [14,93]. This period is also correlated with heightened aggression [11,14,94], yet this subject exhibited no outward aggression towards younger adults present during estrous playbacks on two separate occasions. It is possible this male did not view the two younger individuals as competition and therefore reacted with little to no aggression. Since more females go into estrus and conceive during years of high rainfall and increased food availability [90], it is possible that 2008 provided ample mating opportunities for this high-ranking musth adult, thereby reducing his interest in prerecorded estrous calls.

Curious to monitor this male’s reaction intensity over time and evaluate the occurrence of habituation, we subjected this same musth elephant to further experimental stimuli during our 2010 field season and observed very different results. During this trial, the mature musth adult responded with the highest intensity to estrous calls by searching for and approaching the sound source. Habituation did not seem to be a factor in this male when infrequently exposed to estrous playbacks across years. Rainfall is not likely to be the cause of the different responses observed in this individual across the years, as both years of playback experiments (2008 and 2010) were categorized as wet ([67,68], Etosha National Park rain data archive, unpub. data). Rather, a combination of multiple biological, social, and environmental factors may have influenced decision-making. Despite individual-level complexity, our findings suggest that the motivation to reproduce serves as the main driver of movement patterns and behavioral response in musth males.

### 4.4. Behaviors before and after Estrous Playbacks

In elephant society, mating events and greeting ceremonies of high intensity often arouse physiological reactions in elephants, including defecation [15,61,73,95,96]. During our study, similar reactions were also observed across male elephants, independent of their behavioral reaction score. Defecation rates increased significantly in the time period after the presentation of estrous calls. The association between estrous vocalizations and mating activities most likely roused excitement and interest in musth 4Q males, as well as non-musth elephants in the younger 1Q–3Q age range. Given that non-musth 4Q males generally chose to move away from the estrous call source but still increased in rate of defecation after playbacks, we suspect that this is indicative of a conditioned evolutionary reaction.

Additionally, adult elephants in musth are known to limit their food consumption and can lose a considerable amount of fluids per day in order to communicate their reproductive status through chemosignaling [63,86]. As such, musth adults defecate less frequently than non-musth males. This can limit the capacity of researchers interested in monitoring the physiological responses and conditions of free-ranging musth elephants via non-invasive fecal sampling techniques. Given that estrous calls triggered defecation in the majority of musth males during this study, reproduction-specific bioacoustics may offer a secondary solution to elephant biologists studying musth male physiology and endocrinology.

We anticipated that elephants in musth would increase their display of musth behaviors following playback presentations. Although musth behaviors did elevate in musth 4Q males with a response score of 1, the results were not significant. This may in part be due to our small sample size, or because most musth males were tested without the presence of a competitor or estrous female that often elicits musth displays. Nonetheless, the results of this study show that broadcasting female estrous calls elicits evolutionarily conditioned responses in mature musth elephants that is worth further exploration.

### 4.5. The Spatial Intersection between Male Elephants and Human-Modified Landscapes

Patterns of movement across the greater Etosha ecosystem confirmed that resident male elephants of post-dispersal age do in fact travel through HMLs adjacent to the northern boundary of ENP. Adult elephants #264 and 267 were detected outside of the park on a number of occasions during both the wet and dry seasons, with #264 traveling the farthest north into agricultural HMLs. In addition, adult #264 moved repeatedly between the Mushara waterhole and non-agricultural HMLs northeast of ENP during his three-month musth period. These findings demonstrate a clear overlap in the seasonal and musth-influenced ranges of male ENP elephants and landscapes occupied by humans, demonstrating the need for HEC mitigation tools in this region. Additional empirical evidence of musth elephant movement in relation to HMLs in our study region and others would offer further insights on the extent and need for conflict mitigation that targets elephants in this demographic group.

In localities where HMLs do overlap with musth elephant home ranges, playbacks of estrous calls may be an effective non-invasive tool to aid in HEC mitigation. Instead of chasing musth males back into parks, park rangers may be able to non-invasively lure musth adults back into protected areas by broadcasting estrous calls from speakers mounted on ranger vehicles at distances ranging between 300 m (current study) and 1.2 km [56], depending on the precision of the trajectory needed. As the male approaches the sound source, rangers could then reposition their vehicle towards the desired location (park boundary) and repeat estrous call playbacks until the target elephant is brought to safety. An evaluation of potential habituation in musth adults to one (or several different) estrous vocalizations would be an important next step to confirm this technique’s application. Further experimentation in collaboration with ranger staff in focal locations would facilitate identifying the safest and most realistic protocol for this proposed technique.

### 4.6. Considerations for Future Estrous Playback Studies and Applications

The estrous rumble is a very low-frequency call (F_0_ of 17–26 Hz emitted at up to 115 dB at 1 m from the source) [56,63], and therefore requires specialized equipment for acquiring the appropriate stimuli. Reproducing the stimuli is not as challenging as large-diameter speakers, capable of reproducing low-frequency sounds, and 12V amplifiers are available in most stereo shops in cities throughout Africa. Previous studies have also demonstrated that elephants may respond naturally to acoustic playbacks that lack the lower frequency levels characteristic of certain vocalizations [50], but further exploration of this is needed to confirm possible applications in HEC mitigation.

Additionally, estrous rumbles are given relatively infrequently by females after mating during the peak of estrus [30,52,61,62]. Thus, obtaining a stimulus is the first challenge to overcome. In this playback study and those conducted previously [56,62], recorded estrous vocalizations from a female in Amboseli National Park, Kenya were used [62]. The fact that the calls elicited responses from male elephants in our Namibian study population is encouraging as to the universal potential of the technique in different populations of savanna elephants across Africa.

During a preliminary investigation of our experimental design, we found that there was a slight preference for one of two estrous callers. This may suggest that individual identity, caller age or stage of estrus, and quality of the recording may influence responses. Reaction intensity of male elephants might also improve when presented with estrous calls from resident females from the same population; however, these were not available at the time of our playback trials. We acknowledge that the use of one acoustic exemplar in this study limits our ability to externally validate our findings, though we are confident that the responses observed are interpreted accurately. We suggest that during the next phase of this study, additional estrous rumble stimuli be recorded and tested to gain a better understanding of what specifically influences the intensity of male response.

In Amboseli, Poole found that a male presented with the same stimuli within the same musth period did not respond the second time [63]. Although we did not witness a decrease in reaction intensity in musth 4Q males tested across multiple trials in a given season (Table S6), and instead observed an increase in behavioral response in one male across two seasons, a detailed study of potential habituation would be useful. Nonetheless, if deterrents work even for one season at one location, we believe the effort would still be worthwhile.

Overall, we suggest testing over varying periods of time, using multiple estrous calls from different callers, as well as with different repetition regimes and presentation distances would further delineate the parameters surrounding the maximum efficacy of reproduction-specific acoustic stimuli as a long-term solution to HEC involving male elephants. Ideally, such a study should also deploy subject males with satellite collars so that the duration of any change in behavior can be measured with accuracy. Estrous females also produce a characteristic estrous roar when they are chased by males [60]. This call is more commonly heard, carries over long distances, and is within the audible range. Therefore, recording and playing back estrous roars may be less expensive and logistically simpler to apply as an HEC mitigation tool. Future studies are needed to determine if this particular estrous call offers an alternative or better bioacoustic stimulus that could be used to manipulate the behavior of younger male elephants, as well as those in musth.

## 5. Conclusions

### Future Directions of HEC Mitigation

Determining effective, long-term solutions to HEC has become increasingly challenging as human populations increase and HMLs expand. Many of the non-invasive mitigation tactics currently implemented aim to passively exclude, (e.g., electric fences, trenches, and other physical barriers) or actively deter, (e.g., fires, loud sounds, and chemical repellents) elephants from human settlements [32,46,97]. However, these strategies do not always adequately treat underlying causes of HEC [32] or incorporate elephant life history patterns and behavioral motivations [6,33,34].

Traditional HEC deterrent methods that rely on eliciting a fear response in elephants are often prone to habituation [97], problems of individual recognition, and sex-dependent responses [20]. Elephants are capable of learning and adjusting their behavior rapidly, especially in a social context. As such, mitigation techniques often require continuous modifications to successfully deter elephants over the long term. While recent applications of beehive fences for HEC mitigation have proved extremely effective among several communities in East Africa [53,98], they do not work everywhere and depend on a community’s experience of beekeeping, environmental conditions, as well as the level of bee activity and aggressiveness [54,99,100]. In addition, HEC mitigation often takes place at night, on foot, and can be exceedingly dangerous. The technique we are proposing requires ranger staff working from a vehicle equipped with the necessary playback source, amplifier, and large speaker mounted to the back of the vehicle, and a team capable of carrying out the playback and monitoring the response of the subject(s).

Results from this and earlier studies [56,63] demonstrate the potential of non-invasively influencing the movement of male elephants using stimuli for which they are evolutionarily conditioned to respond. We suggest that such playbacks might be used to lure sexually active males and post-dispersal young males away from human settlements under a prescribed set of circumstances. Furthermore, it has been demonstrated that male elephants are capable of differentiating between the urine of estrus females and those that are unreceptive [101,102]. Natural chemical exudates in African elephants that elicit responses of avoidance or attraction in free-ranging elephants may serve as a useful complementary tool in HEC mitigation, but further exploration is needed [103,104]. As such, further investigation of mitigation strategies that incorporate a combination of elephant-specific chemical and sensory cues may lead to innovative and long-term HEC solutions [105]. Overall, the better we understand male elephant behavior and decision-making in the context of life history and motivational states, the more likely we are to uncover novel mitigation tools for human–elephant coexistence.

## Figures and Tables

**Table 1:** Age class and musth status of male African elephants (*n* = 19) subjected to estrous playback experiments at the Mushara waterhole in 2008. Individuals in the 1Q–3Q age range were grouped together for statistical analyses, whereas all males in the 4Q class were considered fully mature adults and further differentiated by reproductive state (musth or non-musth).

Age Class ^1^	1Q	2Q	3Q	4Q
Relative Age (years)	10–14	15–24	25–34	≥35
Musth	0	0	0	5
Non-musth	2	1	5	6

^1^ Age Class Abbreviations: 1Q = one quarter, 2Q = half, 3Q = three quarter, and 4Q = full.

**Table 2:** Ethogram of focal behaviors observed in male elephants (*n* = 19) during estrous playback experiments. Response score criteria, set on a gradient scale from 0 to 1, were based both on the occurrence and frequency of these behaviors. In the case that certain behaviors were observed in different contexts, both are listed, (e.g., attentiveness and vigilance). In addition, elephants who did not display any of these behaviors in response to playbacks and continued along their movement trajectory were given a score of 0. Further details on the application of these behaviors to response score criteria can be found in Figure 2.

Context	Focal Behaviors	Description
Attentiveness, Vigilance	Freeze	Upon presentation of estrous playbacks, elephant immediately stops all movement, spreads ears, leans toward, and appears to listen to the stimulus.
	Over the Shoulder	Without orienting his body, the elephant glances back over his shoulder in the direction of the hidden speaker. This is often accompanied by the elephant discretely smelling in the direction of the acoustic source with his trunk hovering above the ground.
	Ears Held Out	Elephant spreads ears and appears alert while listening.
	Smell in Direction of Speaker	Elephant smells in the direction of the speaker, either by discretely smelling with trunk hovering above the ground, or using ‘Periscope Trunk,’ with trunk extended high or above their head toward the acoustic stimulus source.
	Orientation	Elephant turns his entire body toward the hidden speaker broadcasting estrous calls in the distance. This behavior is followed by returning to their original position and/or approaching the sound source.
Attraction	Approach	Elephant walks eagerly and intentionally in the direction of the speaker.
	Track	Individual searches for the source of the estrous call, often with a purposeful walk. Elephant is also observed smelling in multiple directions while sweeping his trunk across the ground with ears held out. This behavior is also characterized by frequent repositioning of the elephant’s body in relation to the sound source, including perpendicular and parallel to the object of interest, potentially in attempt to localize the signal.
	Musth Display	Elephant advertises his reproductive status via temporal gland secretions, urine dribbling, ear waving, trunk curling, trunk dragging, musth walking, and tusking the ground.
Social Cohesion	Follow	Elephant moves behind and in the same direction as a conspecific that is often older, higher ranking, and/or socially bonded with him. The individual leading the movement may solicit following behavior through vocal and tactile cues and may also be observed waiting for the follower.
Avoidance	Retreat	Elephant moves quickly in the opposite direction of a perceived threat.

**Table 3:** One-way ANOVA results for the behavioral responses of male African elephants (*n* = 19) subjected to estrous call playbacks in 2008. Pairwise comparisons across three male groups (non-musth 1Q–3Q, non-musth 4Q, and musth 4Q) were conducted using a Tukey’s HSD post hoc test (95% CI) to evaluate between-group differences in behavioral response.

Predictor	Sum of Squares	Df	Mean Squares	*F*	Pr (>*F*)
Male Group	0.957	2	0.478	7.43	0.005 **
Residuals	1.030	16	0.064		
**Post Hoc Comparisons**		**Mean Difference**	**95% Confidence Interval**		**Adjusted *p*-value**
			**Lower**	**Upper**	
Musth 4Q – Non-musth 4Q		0.523	0.126	0.919	0.009 **
Musth 4Q – Non-musth 1Q–3Q		−0.072	−0.445	0.301	0.874
Non-musth 4Q – Non-musth 1Q–3Q		0.451	0.097	0.804	0.012 *

Significance Levels: * means *p* < 0.05 and ** means *p* < 0.01.

## Data Availability

The authors confirm that the behavioral data supporting the findings of this study are available within the article and its supplementary materials online at https://www.mdpi.com/article/10.3390/ani12091162/s1. Suppl R scripts utilized for all statistical analyses are also available at https://github.com/monica-n-sandri/Estrous-Playback-Experiments-with-Male-African-Elephants (accessed on 20 January 2022).

## Supplementary Materials

The following supporting information can be downloaded at https://www.mdpi.com/article/10.3390/ani12091162/s1. Table S1: rater scores of male elephant responses to estrous playbacks for IRR test, Table S2: response scores of 19 males averaged across 26 trials and two raters, Table S3: defecation rates before and after playbacks for all males in our study, Table S4: rate of musth behaviors recorded in all mature musth adults before and after playbacks, Table S5: rate of musth behaviors before and after playbacks recorded in mature musth adults with a response score of one, and Table S6: raw response scores of 19 male elephants across 26 trials and two independent raters. Figure S1: Wilcoxon signed-rank test of the mean rate (±SEM) of musth behaviors observed in musth 4Q elephants with a response score of 1 “before” and “after” estrous playback trials, and Figure S2: movement data of three post-dispersal male elephants within the northeastern region of Etosha National Park during the wet and dry season. In addition, Video S1: a musth elephant’s response to playbacks of an estrous call is available at http://www.utopiascientific.org/Videos/Ele108.html (accessed on 20 January 2022).
