# Supplementary material for: Male African Elephant (Loxodonta africana) Behavioral Responses to Estrous Call Playbacks May Inform Conservation Management Tools
Source: Animals (Basel). 2022 May 1;12(9):1162. doi: 10.3390/ani12091162 (PMC9102362; doi:10.3390/ani12091162)
Supplement: Supplementary file 1 [file animals-12-01162-s001.zip › Animals-1639472-Supplemental Figures.pdf]

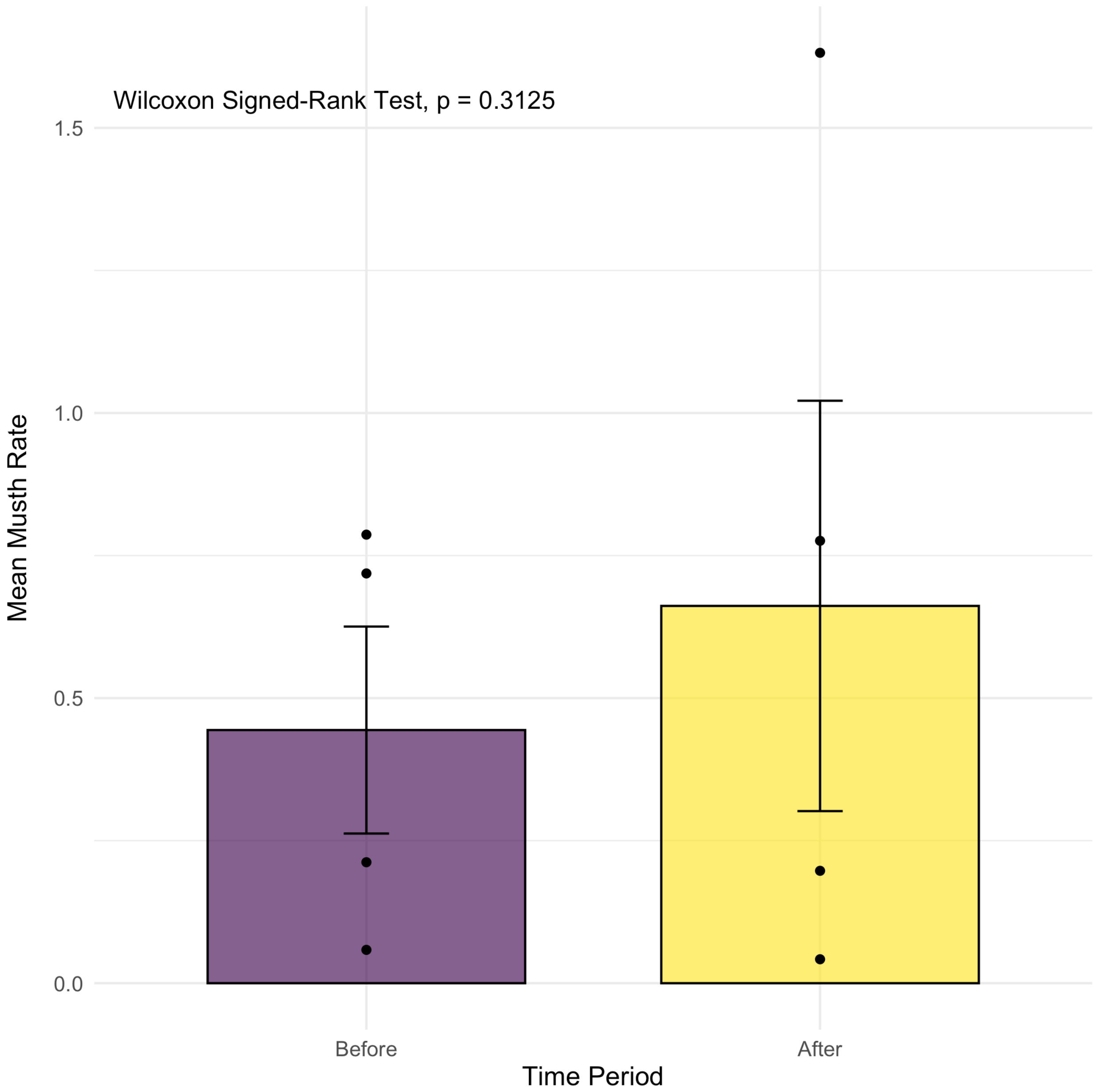

Figure S1. The mean rate (± SEM) of musth behaviors observed in mature musth adult elephants with a response score of 1 (n = 4) "before" and "after" estrous playback trials, regardless of behavioral response score. Observed behaviors that fell under the category of musth and could be counted for this analysis include: ear wave, musth walk, trunk drag, trunk curl, and tusking the ground. Individual elephants are depicted by a single point per time period. An exact Wilcoxon signed-rank test of determined there was no significant difference between musth rates observed before and after playbacks (p = 0.31, effect size r = 0.37).

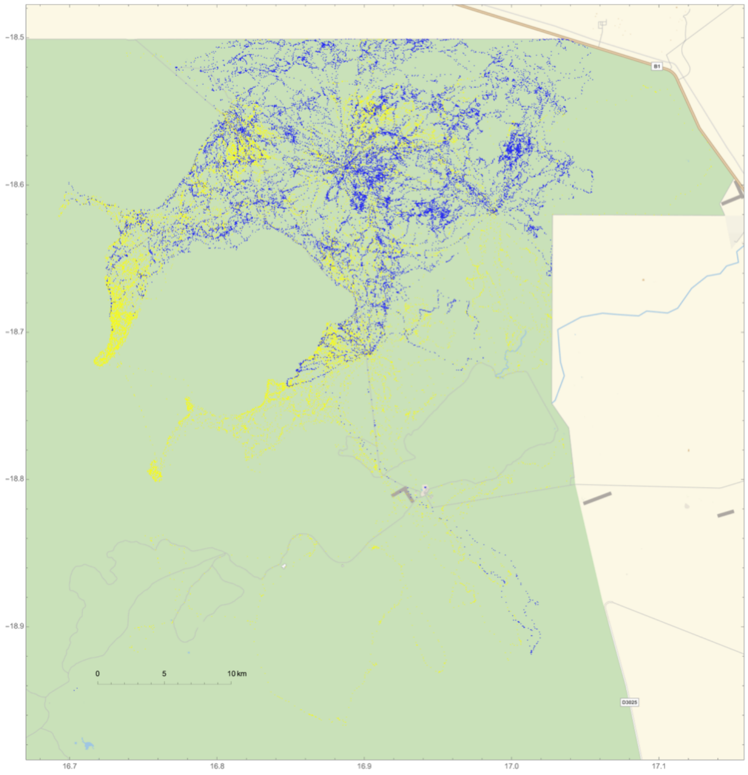

#265

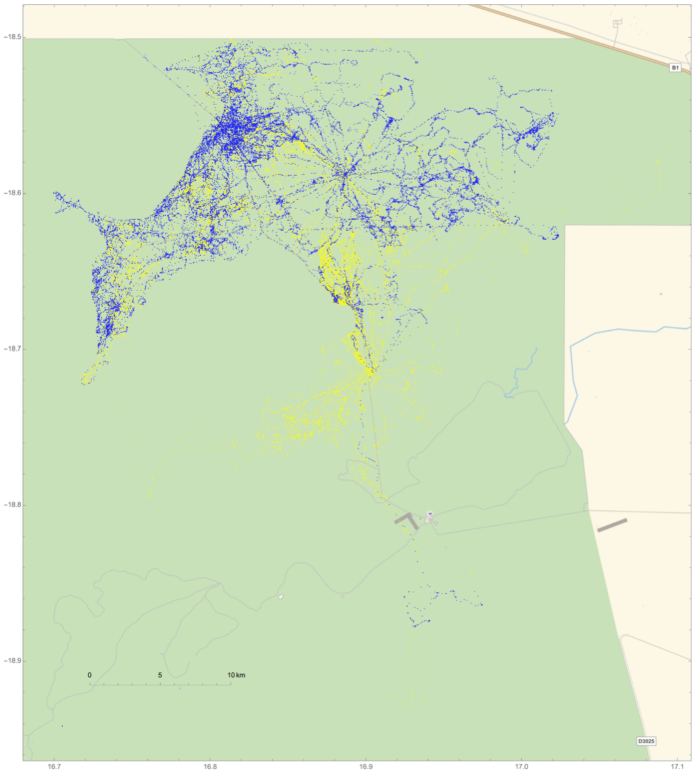

#266

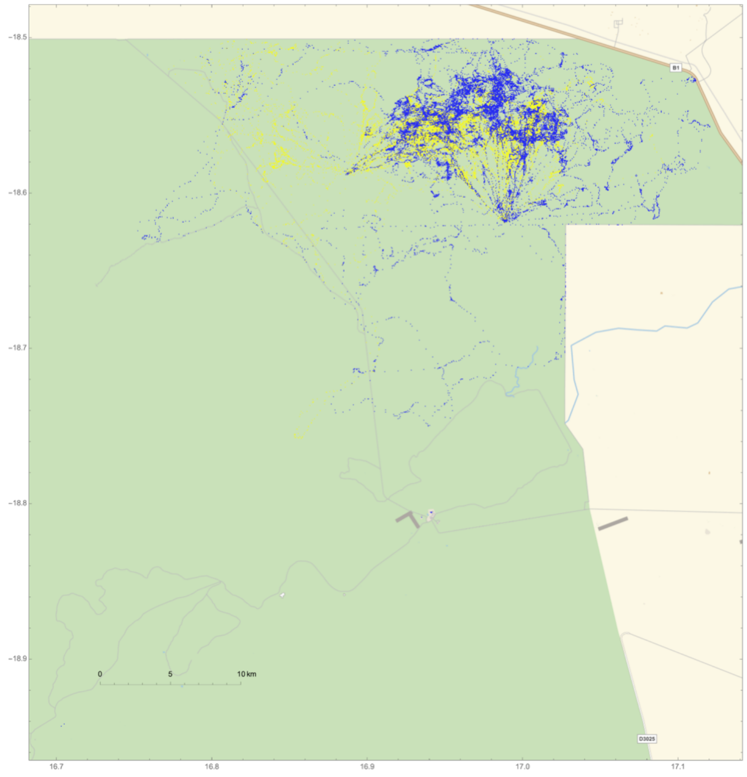

#268

Figure S2. Movement data of post-dispersal male elephants (#265: 2Q age class; #266 and #268: 4Q age class) within the northeastern region of Etosha National Park, Namibia and collected between October 2009 and November 2011 using GPS collars. These devices were programmed to record positional information (longitude and latitude, as shown on the x and y axis, respectively) every 15 minutes. Blue points represent elephant movement during the wet season (May—October), and yellow points showcase movement during the dry season (November—April).
